# Electroporation in Head-and-Neck Cancer: An Innovative Approach with Immunotherapy and Nanotechnology Combination

**DOI:** 10.3390/cancers14215363

**Published:** 2022-10-31

**Authors:** Silvia Pisani, Giulia Bertino, Adriele Prina-Mello, Laura Deborah Locati, Simone Mauramati, Ida Genta, Rossella Dorati, Bice Conti, Marco Benazzo

**Affiliations:** 1 Department of Otorhinolaryngology, Fondazione IRCCS Policlinico San Matteo, Viale Camillo Golgi, 19, 27100 Pavia, Italy; 2 LBCAM, Department of Clinical Medicine, Trinity Translational Medicine Institute, Trinity College Dublin, Dublin 8, Ireland; 3 Centre for Research on Adaptive Nanostructures and Nanodevices (CRANN), Trinity College Dublin, DO2 W085 Dublin, Ireland; 4 Translational Oncology, IRCCS ICS Maugeri, 27100 Pavia, Italy; 5 Department of Internal Medicine and Therapeutics, University of Pavia, 27100 Pavia, Italy; 6 Department of Drug Sciences, University of Pavia, Via Taramelli 12, 27100 Pavia, Italy; 7 Department of Clinical, Surgical, Diagnostic and Pediatric Sciences, University of Pavia, 27100 Pavia, Italy

**Keywords:** electroporation, electrochemotherapy, immunotherapy, nanotechnology, head-and-neck cancer

## Abstract

**Simple Summary:**

This review provides a summary of the head-and-neck squamous cell carcinoma (HNSCC) biological characteristics and its current treatments. Furthermore, insight and outlook on the relationship between electroporation and its implementation (combination with nanotechnology and immunotherapy) in the treatment of H&N cancers are provided.

**Abstract:**

Squamous cell carcinoma is the most common malignancy that arises in the head-and-neck district. Traditional treatment could be insufficient in case of recurrent and/or metastatic cancers; for this reason, more selective and enhanced treatments are in evaluation in preclinical and clinical trials to increase in situ concentration of chemotherapy drugs promoting a selectively antineoplastic activity. Among all cancer treatment types (i.e., surgery, chemotherapy, radiotherapy), electroporation (EP) has emerged as a safe, less invasive, and effective approach for cancer treatment. Reversible EP, using an intensive electric stimulus (i.e., 1000 V/cm) applied for a short time (i.e., 100 μs), determines a localized electric field that temporarily permealizes the tumor cell membranes while maintaining high cell viability, promoting cytoplasm cell uptake of antineoplastic agents such as bleomycin and cisplatin (electrochemotherapy), calcium (Ca^2+^ electroporation), siRNA and plasmid DNA (gene electroporation). The higher intracellular concentration of antineoplastic agents enhances the antineoplastic activity and promotes controlled tumor cell death (apoptosis). As secondary effects, localized EP (i) reduces the capillary blood flow in tumor tissue (“vascular lock”), lowering drug washout, and (ii) stimulates the immune system acting against cancer cells. After years of preclinical development, electrochemotherapy (ECT), in combination with bleomycin or cisplatin, is currently one of the most effective treatments used for cutaneous metastases and primary skin and mucosal cancers that are not amenable to surgery. To reach this clinical evidence, in vitro and in vivo models were preclinically developed for evaluating the efficacy and safety of ECT on different tumor cell lines and animal models to optimize dose and administration routes of drugs, duration, and intensity of the electric field. Improvements in reversible EP efficacy are under evaluation for HNSCC treatment, where the focus is on the development of a combination treatment between EP-enhanced nanotechnology and immunotherapy strategies.

## 1. Head-and-Neck Cancers

Head-and-neck squamous cell carcinoma (HNSCC) is the most common malignancy that arises in the H&N district and is classified as the sixth most common cancer worldwide, with an incidence of 890.000 new cases and 450.000 deaths registered in 2018 [1].

**Figure 1:**
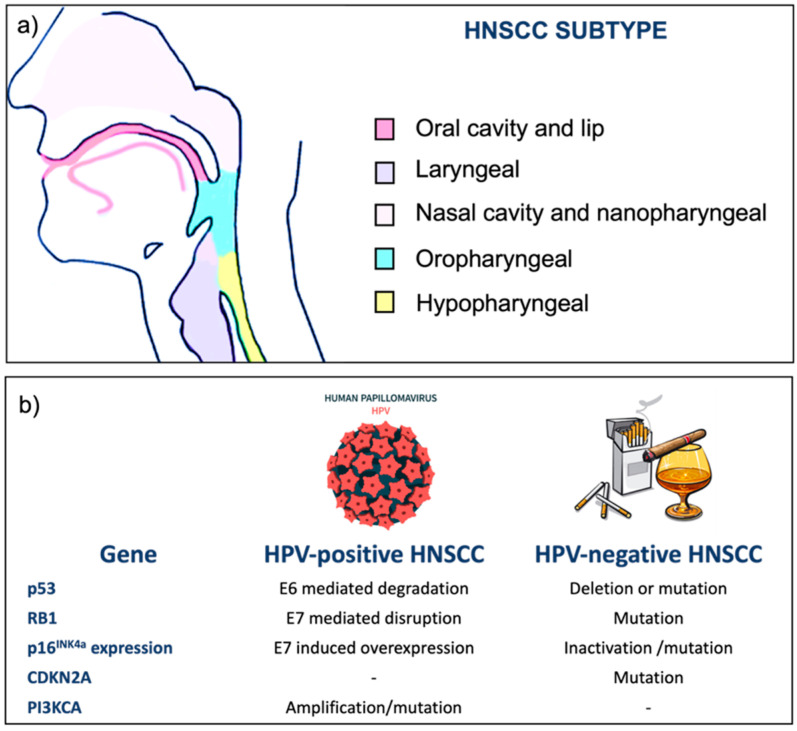
(**a**) Anatomical sites of HNSCC subtype; (**b**) main genetic differences between HPV-positive and HPV-negative HNSCCs.

Squamous cells that surround the mucosal epithelia of the oral cavity, nasopharynx, oropharynx, hypopharynx, and larynx are those most affected by the possibility of the onset of HNSCC. Therefore, squamous cell carcinoma can have origins in different parts of the H&N area such as lips, buccal mucosa, hard palate, tongue, the floor of the mouth and retromolar trigone, palatine tonsils, lingual tonsils, base of the tongue, soft palate, uvula and posterior pharyngeal wall, the bottom part of the throat and extending from the hyoid bone to the cricoid cartilage (Figure 1a) [2].

The main risk factors for HNSCC were registered from epidemiological studies and classified by the International Agency for Research on Cancer (IARC) of the World Health Organization (WHO) [3]. Main exogenous risk factors include tobacco, alcohol consumption, exposure to environmental pollutants, and viral agents’ infection. Human papillomavirus (HPV-16 and HPV-18) is the most common oncogenic viral agent, and human papillomavirus-associated HNSCCs start primarily from the palatine and lingual tonsils of the oropharynx. Instead, HPV-negative HNSCCs begin in the oral cavity, hypopharynx, and larynx. Around 75–85% of HNSCCs are HPV-negative, and they are associated with poor prognosis compared to HPV-positive HNSCCs [4,5]. Moreover, epigenetic alterations such as DNA methylation, histone covalent modifications, chromatin remodeling, and non-coding RNAs resulted involved in HNSCC (HPV-negative), tumor progression, and resistance to traditional therapy [6].

Differences between HPV-positive and HPV-negative HNSCC are also highlighted by distinct gene expression, gene mutation, and immune profiles (Figure 1b). The Cancer Genome Atlas (TCGA) reports whole data of copy number alterations, mutational profiles, and mRNA expression from more than 520 human HNSCC [7]. Compared to other tumor types, HNSCC is more frequently induced by loss of tumor suppressor, consisting of loss of chromosomal regions and multiple genetic alterations [7]. Tumor suppressor protein p16 ^INK4a^ is encoded by the CDKN2A gene and is considered a prognostic factor for positive-HPV oropharyngeal cancer. Clinical practice guidelines for HNSCC use the p16 immunohistochemistry (ICH) test to identify HPV-positive cancers, and in case of positive results, other specific HPV tests are planned to confirm cancer origin [5,8].

Histologically progression of HNSCC follows an ordered series of steps that can be classified depending on specific genes alteration/mutations [2]:

1. Epithelial cell hyperplasia characterized by loss of 9p21 and consequent downregulation of tumor suppressor genes (TSGs) such as CDKN2A.
2. Dysplasia (mild, moderate, severe) marked by loss of 3p21 and 17p13 that regulates p53.
3. In situ carcinoma characterized by loss of 11q13, 13q21, and 14q32.
4. Invasive carcinoma in which is observed loss of 6p, 4q27, and 10q23.
5. Second primary tumors (SPTs) and metastases localized at distinct anatomical sites (esophagus, lungs, skin) can express the same molecular abnormalities of the primary tumor or different markers.

Depending on the HNSCC stage and type, specific guidelines and pharmacologic treatments are approved and used in clinics [5,9,10]. Briefly, first-line treatment includes surgical resection, followed by adjuvant radiation or chemotherapy in addition to radiation (known as chemoradiation or chemoradiotherapy (CRT) depending on the cancer stage and pathological risk factors [2,5]. Suitable treatment strategy should be therefore discussed in a multidisciplinary team including not only surgery and medical oncology but also specialist involved in diagnosis (radiologist, nuclear medical doctor, and pathologist) and in supportive care (nutritionist, pharmacist, researcher, psychologist, physiotherapist, and occupational speech). Innovative treatments, recently approved by U.S. Food and Drug Administration (FDA) and European Medicines Agency (EMA), included monoclonal antibodies for selective immunotherapy depending on the biomarkers overexpressed by tumor in recurrent and/or metastatic HNSCC [11,12].

In general, early-stage diseases in the oral cavity, laryngeal, hypopharyngeal, and p16- negative and p16-positive oropharyngeal cancer are treated with a single-modality approach, radiotherapy (RT), or conservative surgery. RT for first-stage disease (I) requires a dose ranging from 66–70 Gray (Gy) total dose; in advanced disease stage (III-IV), the total dose could be increased to 80.5 Gy cumulative [13].

Minimally invasive surgery techniques, such as robotic surgery and laser microsurgery, are widely used to resect limited tissue portions in early stage disease, preserving organ functionality and improving patient quality of life (QoL) [14]. In the case of locally advanced disease (stage III and IV of oral cavity, larynx, hypopharynx), the standard treatment option could be surgery, sometimes in combination with RT +/- chemotherapy or exclusive CRT. In this case, the surgery requires the complete resection of the diseased tissue and musculoskeletal, vascular, and/or nerve reconstruction to enable the return to the acquired physiological and bodily functionality. Traditionally, organ/tissue reconstruction is performed using autologous materials; for example, the resected esophagus is replaced with an autologous intestine conduit [15]. On the latter, nowadays, improvements in tissue engineering and regenerative medicine are developing synthetic biodegradable and biocompatible engineered scaffolds that are emerging as alternative and useful organ substitutes [16,17]. Some clinical trials are currently underway for the validation of engineered scaffolds also for application in the H&N district (NCT01997437, NCT02949414, NCT01977911, NCT02770209, NCT01242618, and NCT04633928) (www.clinicaltrial.gov, accessed on 28 October 2022).

CRT is also used as standard treatment in a single treatment or in combination with surgery. The most common chemotherapy drugs clinically used are cisplatin, carboplatin, and 5- fluorouracil (5FU) [18] in combination with taxanes (such as Paclitaxel and Docetaxel) [19]. More in detail, Cisplatin (CisPt) is an antineoplastic agent widely used in many cancer therapies. Mechanism of action is based on DNA intercalation and consequent cross-linking that inhibits cell replication inducing apoptosis. Radiotherapy combined with three weekly administrations of 100 mg/m^2^ cisplatin is the accepted therapeutic standard in HNSCC [20].

Bleomycin (BLM) is a cytotoxic glycopeptide antibiotic produced by *Streptomyces verticillus*. Its activity induces DNA strand breaks and superoxide radical production that cleave DNA. After intravenous injection, bleomycin keeps at a high concentration in blood for one hour then it is mainly excreted by the urine. From the pharmacokinetics of BLM, it was shown to have a higher concentration in the skin, peritoneum, and lungs [21]. EMA approved BLM (15 U (USP)/vial) as a powder for solution for injection (Local/Intratumoral injections), and its use is also allowed in combination with anti-cancer drugs or in combination with radiation. Regarding the indication in head-and-neck cancer, there is still a role for bleomycin in the (neoadjuvant) treatment of the disease [22,23,24].

Despite this, chemotherapeutics are effective in their treatment; they are impacted by many severe side effects (e.g., pulmonitis, pulmonary fibrosis, stomatitis and skin changes, fatigue, hair loss, easy bruising and bleeding, infection, anemia, etc.), which reduce the patients QoL.

The increasing study focused on HSNCC molecular biology identified a considerable number of molecular biomarkers useful to be used as a target for more selective and targeted therapies such as immunotherapy. Among all, EGFR, CD44, CD133, and ALDH1 are overexpressed by HNSCC and cancer stem cells (CSCs) and are associated with prognostic significance [25].

The identified molecular biomarkers of HNSCC and CSCs are reported and explained in Table 1.

EGFR, overexpressed in 80–90% of HNSCC tumors, is associated with low overall survival (OS). Instead, high levels of CD44 are associated with metastasis and poor prognosis. High levels of ALDH1 expression or activity are associated with self-renewal, invasion, and higher metastasization in HNSCC. Moreover, analysis of HNSCC showed that 80% of ALDH1+ cells are closer (<100 μm) to a blood vessel, suggesting that CSCs reside primarily in perivascular niches [33].

Over-expression of PD-L1 (PD-L1+) has been recorded in 24–49% of melanoma cancer cases and is related to increased faster growth and poor overall survival (OS). PD-L1 is overexpressed in about 80% of cases of varying degrees in gastro-esophageal, colorectal, and bile duct carcinomas and is usually associated with a poor prognosis [34].

Other CSCs markers (OCT3-OCT4-SOX2 and NANOG) are identified, and their levels are correlated with tumor grade in oral cancer [35].

Immunotherapy for HNSCC is an innovative and valuable treatment without the potentially devastating side effects of conventional treatments. Basically, immunotherapy works by helping the immune system recognize and attack cancer cells. Using molecular biomarkers as a target, a monoclonal antibody was used to selectively target cancer cells or protein on cells of the immune system, ignoring healthy cells. Incorporation of prognostic and predictive biomarkers into clinical management may overcome obstacles to targeted therapies and enable prolonged survival.

Monoclonal antibodies currently used in the treatment of HNSCCs are reported in Table 2.

Cetuximab (Erbitux) is a chimeric monoclonal antibody (mAb) of the immunoglobulin G1 (IgG1) class. The affinity for EGFR is approximately 5 to 10-fold higher compared to endogenous ligands, so it is able to block the binding of these ligands, resulting in inhibition of the receptor function.

In 2006, Cetuximab was FDA-approved for H&N cancer treatment either for local/regional advanced SCC in combination with RT or as a monotherapy (400 mg IV 120 min/1° week, followed by 250 mg/m^2^) for recurrent or metastatic HNSCC progressing after platinum-based therapy. In 2011, FDA approved Cetuximab for late-stage HNSCC in combination with chemotherapy (recurrent locoregional disease or metastatic HNSCC in combination with platinum-based therapy and with Fluorouracil) [39].

Cetuximab induces EGFR internalization, which can lead to its downregulation [40]. Another mechanism of action identified for cetuximab is to induce antibody-dependent cell cytotoxicity (ADCC) through Fcγ receptors on immune effector cells [41,42]. ADCC is a set of mechanisms that target appropriate subclasses of cells coated with IgG antibodies (IgG1 in humans) to be attacked by cell-to-cell cytolysis performed by FcRIIIA (CD16A) expressing immune cells [43]. In cancer therapy, ADCC is exploited by antibodies that selectively recognize surface proteins on malignant cells. Lattanzio et al. investigated the impact of baseline ADCC on the patients’ outcomes presenting locally advanced HNSCC treated with cetuximab and radiotherapy [44]. In this study, patients showing a high baseline of both ADCC and EGFR have a significantly higher probability of achieving a complete response and a long OS compared to other patients.

Pembrolizumab (Keytruda) is a humanized IgG4 isotype antibody. In 2019, Pembrolizumab was approved by FDA as first-line treatment or in combination with platinum and FU for patients with metastatic or unresectable recurrent HNSCC. Moreover, Pembrolizumab monotherapy is indicated by EMA for first-line treatment of patients with metastatic or unresectable, recurrent HNSCC whose tumors express programmed death ligand 1 (PD-L1- combined positive score (CPS) ≥1).

**Figure 2:**
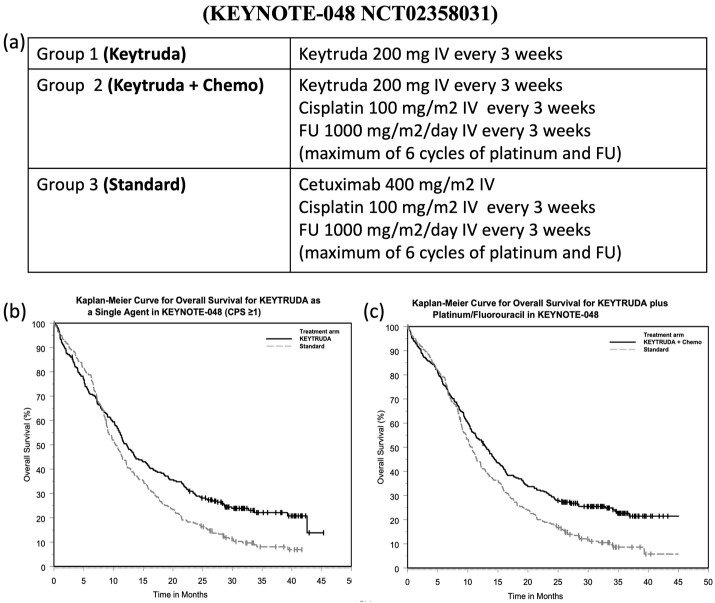
KEYNOTE-048, (**a**) randomized groups and treatments used; (**b**) Keytruda as a single agent and standard (Cetuximab + Cisplatin + 5-Fluorouracil); (**c**) Keytruda + chemo and standard (Cetuximab + Cisplatin + 5-Fluorouracil [37].

The Pembrolizumab dose recommended for HNSCC is 200 mg administered as an intravenous (I.V.) infusion (30 min infusion time/every 3 weeks). Therapy can be modified if disease progression keeps going, unacceptable toxicity is registered, or up to 24 months in patients are not highlighted disease progression. The Pembrolizumab efficacy was investigated in a randomized, multicenter, open-label, active-controlled trial conducted on 882 patients with metastatic HNSCC and the one, which did not have previously received systemic therapy for metastatic disease, or with recurrent disease considered incurable by local therapies (clinical trial reference: KEYNOTE-048 NCT02358031) as shown in Figure 2a–c [37,45].

This emerging PD-1/PD-L1 blockade immunotherapy exhibited more satisfactory curative effects, and lower toxicity for patients with advanced HNSCC compared to standard (Cetuximab + Chemo) treatment [46]. Management of recurrent and/or metastatic HNSCC (50% of patients with locally advanced HNSCC will recur after primary treatment) that are not amenable to surgery and show PD-L1 expression are currently treated with Pembrolizumab + cisplatin and FU, showing an improvement in the overall survival (OS).

In case of recurrent and/or metastatic HNSCC not expressing PD-L1, Cetuximab combined with platinum-based therapy is the standard of care. Instead, in the case of HNSCC progression within 6 months since the last platinum therapy, Nivolumab is both FDA- and EMA-approved therapy [5].

Recurrent and metastatic HNSCC still remain problematic to treat. The most common metastatic sites are the lung, bone, liver, and skin [47]. Traditional therapies are not able to eradicate all cancerogenic cells, and in some cases, surgery is impossible. More selective and enhanced treatment should be used to increase chemotherapy drug concentration only at the cancer site, enhancing antineoplastic activity.

**Figure 3:**
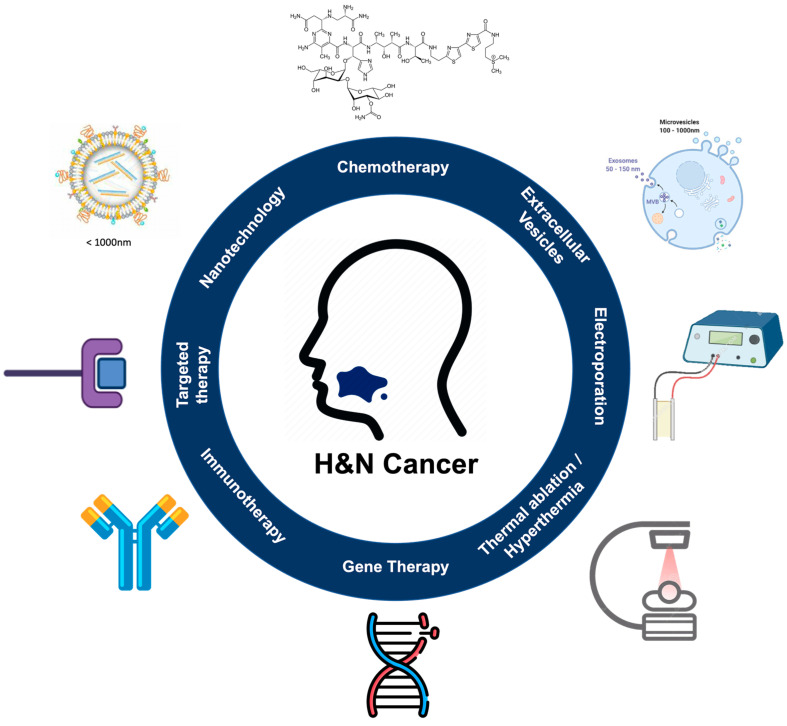
Innovative approaches for H&N cancer treatment including cell and gene therapies, physical and chemically triggered localized stimuli, antibody and immuno- therapies, extracellular vesicles, and nanomedicines (created with BioRender—www.BioRender.com, accessed on 28 Octorber 2022).

Innovative approaches for recurrent H&N cancer treatment are under evaluation in preclinical and clinical trials; among these, we include extracellular vesicles, thermal ablation/hyperthermia, gene therapy, and nano-immunotherapy as summarized in Figure 3 [11,48,49,50,51,52]. These treatment strategies can come as combined interventions to obtain a synergic and enhanced anticancer effect.

In this review, we expand on the application of reversible electroporation (electrochemotherapy, gene electroporation, calcium electroporation) as HNSCC treatment and its possible use with immunotherapy and/or nanotechnology-based strategies.

## 2. Electroporation

Electroporation/Electropermeabilization (EP) results in the application of a localized electric field able to increase the permealization of molecules into cell membranes. Due to the physically triggered phenomenon, the cell membrane can induce the temporary depolarization of the voltage-gated channels, which subsequent increase the cell permeability by hydrophilic pores formation (≈23 nm radius) [53]. EP of cells in vitro can be used for the introduction of DNA, enzymes, antibodies, and other biochemical reagents. However, EP has begun to be investigated to enhance cancer tumor chemotherapy, transdermal drug delivery, non-invasive sampling for biochemical measurement, and localized in situ gene therapy [54,55].

Briefly, when an electrical field pulse (EFPs) is applied, a transient change in permeability and electrical conductivity in the cell’s membranes (phospholipid bilayer membranes thickness of h ≈ 3 to 7 × 10^−9^ m) is induced. EFP must be applied with short times and in a high electrical field in order to achieve suitable membrane permealization able to permit passage of any molecules through cell membrane pores. Essentially, the driving force is the physical interaction of electric fields with two deformable materials with different dielectric constants (K): such as lipids (l), with K_l_≈ 2–3 and aqueous electrolytes, with K_e_ ≈ = 70–80 = K_w_, where _e_ denotes electrolyte and _w_ water [56,57]. At this point, an accumulation of charges due to ions migration occurs, and the cell membranes undergo a rearrangement in their morphology after exceeding a critical threshold causing the rapid creation of aqueous pathways through lipid-containing barriers in cells and tissue (transient hydrophilic pores are formed). Electroporation occurring when the cell transmembrane voltage, (Vm), reaches values (0.5–1 V) much higher than the normal values of “resting potential” (≈−0.1 V) developed for living cells [56,58]. The resting potential is important for two events: the threshold required for permeabilization and the electroporation steps. Considering that cells are physiologically negatively charged, permeabilization happens first of all in the area of the cell facing towards the electrode with a positive charge; because in this area, the membrane capacitance is the first to exceed when an external field is applied. Subsequently, the portion of the cell facing the negative electrode is electroporated. Other aspects, such as the continued permeabilization (area of the membrane which is permeabilized) on the area facing the positive electrode, can be modulated by pulse amplitude, i.e., higher pulse amplitude enhance the diffusion area through the cell membrane. Instead, the permeabilization degree can be controlled by the pulse duration and pulse number), i.e., a longer pulse enhances the perturbation of the membrane in the treated area [59]. Therefore, it was seen that the cell membrane that is most sensitive to the effect of the electric field is the one closest to the positive electrode, but instead, the degree of permeabilization is greater for the membrane facing the negative electrode [60]. Thus, larger molecules will be able to diffuse into the cell through the membrane facing the negative electrode, but the area over which diffusion can take place is larger towards the positive electrode [61].

Electric field parameters that control membrane electroporation are electric field strength, pulse duration (nanosecond, microsecond, or millisecond), number of applied pulses, and pulse frequency. Usually, nanosecond electrical field pulses (NsEFPs) use a field strength of kV/cm (20 kV/cm and greater) while micro-and millisecond use 100–1000 V/cm [57].

The transmembrane potential induced in a cell by an external field, such as EFP, is usually described by the Schwan equation (Equation (1))

(1) ∆Vm= f ×E(t)×R× Cosφ

where Vm is the transmembrane potential, “f“ is a cell-shape factor (usually 1.5 for spherical cells), “E(t)” is the applied external electric field for a determined time, “R” is the cell radius,” φ” is the polar angle between the direction of E and the specific location on the membrane [62]. Other parameters that can influence membrane electroporation are the composition of the electroporation buffer, temperature, and cells’ intrinsic properties (size, type, shape, density, and adhesion) [63].

One aspect is that with a smaller cell radius, a higher external electric field is needed to achieve suitable permeabilization. The electric fields required for mammalian cells’ permeabilization are much lower than those required to permeabilize, e.g., bacteria. It is also evident that, e.g., mitochondria or other intracellular organelles will not be permeabilized by the same electric fields used to permeabilize the cell membrane.

Depending on pulse exposing time and electric field intensity, three different types of EP exist [64]:

1. Reversible EP: The electric field is sufficient enough (≈1 kV/cm) to exceed the critical threshold, but the cell is still able to return to its initial state (resting potential). Due to its reversible pore formation in the microsecond time frame, membrane resealing happens over a range of minutes. In an in vivo experiment performed on mouse skeletal muscle tissue, it was found that a 63% resealing time (s) required approximately 9 min [65]. Due to it is not irreversible destructive action, this type of EP is used to insert molecules in the intracellular environment (i.e., Electrochemotherapy, Genetic transfer, and Calcium electroporation).
2. Irreversible EP: The electric field is extremely high (>2–5 kV/cm), and the number of pores created induces cell osmotic imbalance or homeostasis loss, resulting in cell necrosis and death (i.e., non-thermal tissue ablation) [66]. Cell death due to irreversible electroporation is a function of electric field strength and pulse number [67].
3. Thermal irreversible EP: Electric field intensity or exposing time is so high that Joule heating is observed (≈10 kV/cm and T° > 50 °C) [68].

Focusing on the effects of reversible EP, EFP causes various effects at different levels. At the membrane level, pore formation (hydrophilic openings) is achieved when ΔVm reaches the threshold causing an electric breakdown of the cell membrane and lipids reorientation [69]. Pore formation and transport through them are conditioned by EFP and EP type. However, pore formation is a stochastic event, and cells can modify their size and shape under electric field stimulation. Moreover, cell type and medium (conductivity, osmolarity, and solutes) can affect EP results.

Instead, at the tissue level, a strong EFP causes highly inhomogeneous EP in multicellular tissue due to differences in cells, the presence of vascularization, and gap junction between cells that made space causing anisotropic electrical properties [70]. To improve EFP homogeneity, a proper design of the EP electrode positioning should be carried out to maximize the targeted effects on tissue and cells [53,71]. Moreover, clinical application demonstrated that EP has two other main effects that can contribute to increasing cancer treatment (i.e., cutaneous, mucosal) efficacy [72]. First of all, it causes a local “vascular lock”, because, during electroporation, the perfusion of the treated tissue is blocked as a result of the vasoconstrictor reflex (vasoconstriction of blood vessels), inducing a hypoxic effect [73]. A differential effect between normal vs. tumor vessels was demonstrated resulting from the application of EP. In fact, EP has a selective vascular disrupting action on tumors by destructing small tumor blood vessels (anti-angiogenic effect) without affecting the larger normal blood vessels surrounding the tumor. This effect is amplified when EP is combined with cytotoxic drugs (electrochemotherapy), also increasing the residence time of the molecules in the treated site [74]. Moreover, in tumor blood vessels, a transient vascular constriction induced by EP prevents further bleeding and even stops previous bleeding in the case of hemorrhagic nodules [73,74].

The second important event correlated to EP application is the local activation of immune system cells causing immune response stimulation and immune cytokines production [75,76]. During EP, the uptake of extracellular proteins happens, and consequently, intracellular proteins escape into the extracellular milieu, acting as a source of damage-associated molecular patterns (DAMPs) that potentially induce an immunogenic response [77]. Moreover, under local electric stimulation, it has been reported in vitro that macrophages polarization in M1(pro-inflammatory macrophages) or M2 (anti-inflammatory macrophages) can be modulated; as well as migration, proliferation, and cytokines production from T-cells can be controlled by EFP application [75,78]. In one other work, Arnold et al. showed that exogenous electrical fields affected the migration, proliferation, and cytokine production of T cells. They demonstrated that human primary T cells migrate directionally to the cathode in low-strength (50–150 mV/mm) electric fields [78]. This immunological stimulation induced by the application of the electric field has a synergistic effect, together with the chemotherapy drug and the vascular lock, in the eradication of the tumor. In fact, electrical stimulation is capable of promoting both cell differentiation and cell death in human cancer environment [79].

### 2.1. Electrochemotherapy (ECT)

The electrochemotherapy technique uses the physical principle of reversible EP to accelerate penetration into the intracellular environment of non-permeant hydrophilic anticancer drug or low permeant anticancer drug (i.e., CisPt) [69]. The membrane/tissue effects mentioned above for EP help chemotherapy drugs to enhance their activity. Membrane poration enhances drugs intracellular concentration increasing their local cytotoxicity (up to 300–700 fold for BLM and 12–70 for cisplatin) [80]. Vascular lock entraps drugs in the tumor site and slows down their washout. Finally, the effect of tumor cell disruption during EP is enhanced during ECT, which induces greater recruitment of antigen-presenting cells (APCs) and release of damage-associated molecular patterns (DAMPs) that, by activating immune cells interacting with pattern recognition receptors (PRRs), act synergistically in fighting against cancer exploiting immune-response [75,81]. By knowing these, mathematical models and optimization techniques have enabled more effective ECT design and electrodes positioning when considering the effect of tissue inhomogeneity on electric field distribution and, consequently, outcome [82].

ECT is becoming a popular operating procedure for many cancer types, including H&N, that do not respond to other first-choice treatments (surgical excision, chemotherapy, radiotherapy). However, it is not still approved for cancer treatment. Indeed, the advantage of this technique is that the physico-chemical principle on which it works (membrane permealization, vascular lock, and immune-stimulation) could be applied to all tumor types (skin or subcutaneous tissue) [83]. Furthermore, compared to traditional chemotherapy, ECT employs lower dosages of chemotherapeutic drugs and can be used as a strategy to avoid drug resistance [84]. Studies reported that a faster and more efficient reduction of tumor size was performed by ECT compared to standard chemotherapy for both cutaneous and subcutaneous tumors [85,86,87].

Comprehensive ECT guidelines were published in 2006 by the European Standard Operating Procedures in Electrochemotherapy (ESOPE) and made a decisive contribution to the standardization of ECT procedures in clinics and in oncology treatment [88,89,90].

In the pre-treatment examination, patients were selected following specific criteria and medical history, which were taken into account together with the presence of allergy or hypersensitivity to the candidate drugs. Tumor entities, as individual units for treatment, are evaluated by imaging (Magnetic Resonance Imaging, Computerized Tomography, Positron Emission Tomography) in terms of size and numbers in order to set up a suitable course for treatment and Standard Operating Procedures (SOP). Identified tumor units are calculated in their volume with the envelope ellipse formula as commonly used in the SOP (Equation (2)) [88,91].

(2) $$V=ab^{2}\pi /6$$

where “*a*” is tumor length, and “*b*” is tumor width.

Depending on tumor size and count, the type of anesthesia and drug administration are decided; for example, tumor that are ≤3 cm and less than 7 are usually treated with local anesthesia (LA) and intra-tumor (I.T.) drug injection. Instead, for tumor >3 cm and in number higher than 7, general anesthesia and intravenous (I.V.) drug injection is preferred.

Drug dose are usually 15,000 IU/m^2^ (I.V) and 1000 IU/m^2^ (I.T) for BLM and 2 mg/ml for CisPt (only I.T.). SOP for ECT in the treatment of mucosal cancer (i.g. oral cavity), cutaneous primary and secondary tumors (metastases derived from HNSCC, malignant melanoma, basal cell carcinoma, breast, and salivary gland adenocarcinoma) were established as eight pulses of 100 μs long at appropriate voltages (1 kV/cm) and frequency 5 kHz with more suitable electrodes depending on tumor location [88].

Different types of electrodes can be selected depending on lesion location and size; for an effective ECT, electrode coverage should be the entire tumor surface. Electrodes that can be used with an electroporator apparatus (i.e., Cliniporator^TM^, IGEA, Carpi, Italy) are finger electrodes and plate electrodes for small and superficial tumor nodules or needle electrodes for deeper and thicker tumor nodules. Among the different types of electrode needles, the two parallel arrays of needle can be selected (4 mm gap between needle) for small nodules and a hexagonal array for tumor nodules bigger than 1 cm [89]. Other electrode types were designed and patented to be more performing and better reach the tumor lesions [92]. Campana et al. studied the effect of electrode position and electric field distribution for non-parallel needles in ECT [93,94]. Their work demonstrated that needle inclination (higher than α = 30° angles inclination) affects the homogeneity of field distribution and is strongly recommended to avoid in living tissue. Moreover, improper insertion of electrodes in tissues could cause local skin burns [95].

Usually, follow-up is programmed 4 weeks after ECT treatment and documented with measurement of tumor size, pictures, and report of pain. In case, further treatment can be performed at least 4 weeks after the first one if BLM (I.V.) was used; otherwise, for I.T. treated lesion, further treatment can be applied whenever needed [88].

European Research on Electrochemotherapy in Head and Neck cancer (EURECA project) reported results of the application of ECT in the treatment of mucosal cancers. The study is supervised by the International Network for Sharing Practice in Electrochemotherapy (INSPECT) and included trials from six European institutions [96,97]. ECT in combination with BLM (I.V. 15,000 IU/m^2^) was used for the treatment of recurrent and/or metastatic H&N cancer using ESOPE guidelines [98]. Patients (n = 43) treated were followed during post-treatment, and tumor response, side effects, and pain were evaluated. The overall objective response after 12 months was 56%, and in 7% of patients, a long-term complete remission was observed. Results indicate that BLM-ECT is a valid treatment for untreatable recurrent H&N mucosal cancer [96].

**Figure 4:**
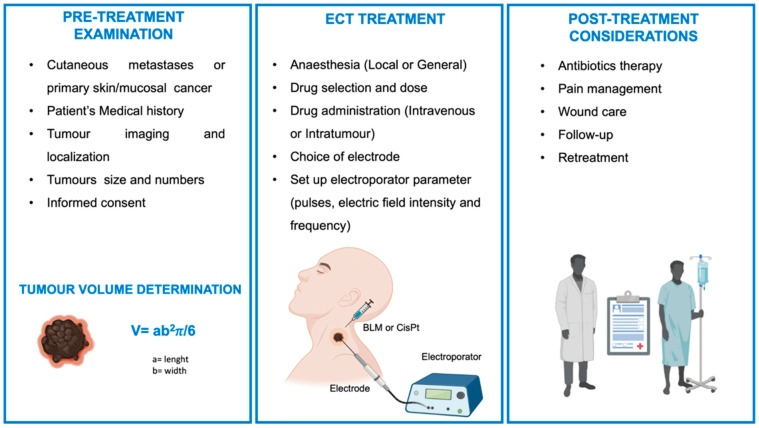
Overview of Standard Operating Procedure (SOP) for Electrochemotherapy (ECT) in the treatment of cutaneous primary and secondary tumors and mucosal cancer (created with BioRender—www.BioRender.com accessed on 28 Octorber 2022).

Other main clinical aspects to be considered in ECT treatment of cutaneous primary and secondary tumors and mucosal cancer are reported in the literature [89,96,99,100] and summarized in Figure 4. Moreover, clinical trials are ongoing to evaluate ECT efficacy and validate further SOP for solid tumors such as the liver, pancreas, and lung [101,102,103,104].

However, some possible adverse effects related to ECT treatment should be mentioned, such as pain at the site of application of the electrodes, redness and swelling in the treated area, muscle contraction, nausea, skin breakdown (rash and mild scarring), and rarely even infections at the application site. It was reported by Landström et al. that ECT in the head-and-neck region may have some limitations related to the onset of lethal bleeding, osteoradionecrosis, and fistula [105].

### 2.2. Gene Electroporation (GE)

Cancer gene therapy is based on gene transfection (insertion of new genetic material into the cell). New DNA introduced is then “transcribed” to make mRNA able to encode a specific protein made through the translation process. Gene therapy can be used to obtain different actions: “corrective”, “cytoreductive”, or “immunomodulatory” [106].

Gene therapy can be used in recurrent HNSCC and localized distant metastatic disease treatments. Many clinical trials are ongoing to validate the safety of gene therapy in HNSCC; for instance, trials no. NCT00009841 or to demonstrate the clinical efficacy of gene therapy when combined with chemotherapy or radiation therapy, under trial no. NCT00017173 [107].

Brezar et al. combined radiotherapy (RT) with gene electroporation (GE) for the delivery of plasmid encoding shRNA against melanoma cell adhesion molecule (MCAM) [108]. Gene therapy goals were to achieve (i) a vascular-targeted effect mediated by the silencing of MCAM and (ii) an immunological effect mediated by the presence of plasmid DNA in the cytosol-activating DNA sensors. Complete antitumor effectiveness using combined therapy (RT + GE) was achieved in immunogenic B16F10 melanoma (81%) while in less immunogenic TS/A carcinoma was reached 27% of complete responses. Moreover, a significant increase in infiltrating immune cells and a radio-sensitization effect was observed in both radioresistant tumor models, while the expression of IL-12 and TNF-α (proinflammatory cytokines of mainly innate immunity) was determined preferentially in the melanoma cancer model. Furthermore, the outcome of the combined modality treatment response of tumors seemed to depend on tumor immunogenicity [109].

Sedlar et al. showed increased efficacy of cisplatin-ECT adding intramuscular interleukin-12 (IL-12) gene electro-transfer [110]. They used murine sarcoma (SA-1) and carcinoma (TS/A) models to test techniques combination and showed that intramuscular IL-12 gene electrotransfer increased the log cell kill in both tumor models, potentiating the specific tumor growth delay by a factor of 1.8–2 and increasing tumor cure rate by approximately 20%. Adil Daud et al. used plasmid IL-12 electroporation (six 100-μs pulses at a 1300-V/cm electric field through a six-electrode array) in patients (n = 24) with metastatic melanoma. Post-treatment biopsies revealed an increase in IL-12 protein levels and higher tumor necrosis (52% showed partial response) and lymphocytic infiltration, demonstrating that IL-12 in combination with electroporation was safe to use but also effective and reproducible [111].

Not only has IL-12 immune cytokine shown to be effective for electroporation-based cancer therapies, but also other genes expressing cytokines (IL-18, IL-33, IL-15, IFn-α, and IFn-γ) were tested in combination with EP to promote the production of T cells and Th1 cells differentiation, increasing antigen presentation and recruitment of dendritic cells [112].

GE was studied in a clinical trial for a safety study of the HPV DNA Vaccine (pNGVL4a-CRT/E7) to treat H&N Cancer Patients (trial no. NCT01493154). In this study HPV DNA vaccine was injected using an electroporation device (TriGridTM Delivery System made by Ichor Medical Systems, San Diego, CA, USA), and the vaccine’s ability to help the body’s immune system to recognize HPV-positive HNSCC was evaluated. They proved that electroporation is a safe, tolerable, and promising method for the delivery of the HPV DNA vaccine, and it should be considered for DNA vaccine delivery in human clinical protocols [113].

Another clinical trial was performed to test HPV-specific immunotherapy on HPV-positive HNSCC patients. DNA vaccine (INO-3112) was delivered by EP (CELLECTRA™-5P, INOVIO Pharmaceuticals) (trial no. NCT02163057). Compared to other techniques (conventional intramuscular injection and epidermal gene gun-mediated particle delivery), electroporation-mediated intramuscular delivery generated the highest number of E7-specific cytotoxic CD8+ T cells, which correlated to improved outcomes in the treatment of HPV-positive HNSCC. Moreover, DNA-vaccine + EP resulted in significantly higher levels of circulating protein which likely enhances calreticulin’s role as a local tumor anti-angiogenesis agent. The study supported further development of EP as a vaccine/gene delivery technique to enhance immunogenicity, particularly for diseases in which traditional vaccination approaches are ineffective [114].

### 2.3. Calcium Electroporation

Calcium (Ca^2+^) is an ion normally present in cells at a physiologic intracellular concentration of 10^−7^ mol/L. It is involved in cell apoptosis, muscle contraction, gene transcription, metabolism, and other functions [115]. In the cells, the endoplasmic reticulum (ER), the sarcoplasmic reticulum (SR, in muscle cells), and mitochondria act as a calcium ions resource. Calcium is pumped into the ER and SR through the sarco-endoplasmic reticulum calcium ATPase (SERCA) [116]. An increase in intracellular Ca^2+^ concentration above the mentioned physiologic values, can lead to toxic effects since it causes ATP depletion and cell necrosis. Combining EP with Ca^2+^ is possible to increase and speed up intracellular ion concentration in cancer cells. For this reason, Calcium electroporation (Ca^2+^-EP) is emerging as a new cancer treatment able to induce cell necrosis by an increase in calcium intracellular influx [117].

The effective anticancer activity of Ca^2+^-EP was tested both in vitro and in vivo, and it demonstrated effective cell necrosis [118,119]. Moreover, it was highlighted that Ca^2+^-EP has a higher effect on tumor cells compared to healthy cells, confirming that healthy tissue surrounding the tumor is less affected by EP treatment. This mechanism can be explained by the fact that the calcium pathway is often modified in cancer cells compared to normal healthy cells. Calcium channels, pumps, and exchangers are present in malignant cells as well as normal cells; however, their expression, localization, and/or activity can be altered (decreased expression of SERCA2 and SERCA3) [120]. These modifications caused a reduced calcium transport from the cytosol to ER in cancer cells.

Planshke et al. performed a clinical phase I study focused on Ca^2+^-EP for recurrent H&N cancer (trial no. NCT03051269). More in detail, they tested the safety of calcium electroporation on mucosal, head-and-neck cancers [117]. The patients selected (n = 6) for the study showed reoccurrence after surgery and RT, thus not indicated for surgery, and with poor tolerance to palliative chemotherapy. A ca2+ dose of 0.225 mmol/ml (or 9 mg/ml) was decided considering tumor volume (calculated according to ESOPE guidelines), including a safety margin of 1 cm tissue surrounding the tumor was treated [89]. EP was performed using a Cliniporator (model EPS02, IGEA, Carpi, Italy) equipped with finger-electrode with linear array needles (10 mm long), set up with the following values of pulse (0.1 msec) intensity (1 kV/cm) and frequency (1 Hz or 5000 Hz).

Data collected demonstrated that Ca^2+^-EP was safe and did not cause hypercalcemia, cardiac arrhythmias, or other severe side effects. Objective tumor responses were observed in three of the six treated patients, with one patient in complete clinical remission one year after treatment [117].

Vissing et al. performed a non-randomized phase II clinical trial (trial no. NCT04225767) to validate a design protocol and investigate tumor response to (Ca^2+^-EP) in cutaneous tumor [121]. Promising results were obtained using EP set up with 220 mmol/L Ca^2+^ with eight pulses of 0.1 ms duration, amplitude 1 kV/cm at a frequency of 1 kHz, where the complete response of 66% patient panel was shown up to 2 months after treatment.

Falk et al., in a randomized double-blinded phase II study (NCT01941901), evaluated Ca^2+^-EP for treatment of cutaneous metastasis from the tumor histology (cutaneous metastasis occurs in 9% of all cancer patients) and compared the results with standard ECT [119,122]. Ca^2+^ dose was defined as 9 mg/ml (220 mmol/L) while BLM was fixed at 1000 IU/ml, and injected volume used for both treatments was 0.5 ml/cm^3^ tumor volume. EP was performed with a linear array electrode; eight pulses of 0.1 ms duration and 400 V at a frequency of 5 kHz were delivered using a square wave pulse generator (Cliniporator^TM^, IGEA, Italy). The data achieved after 6 months of treatment showed that Ca^2+^-EP obtained an objective response of 72% (i.e., 66% complete response, 5% partial response) while ECT achieved 84% (i.e., 68% complete response and 15% partial response). This study confirmed that Ca^2+^-EP is feasible in clinical settings with minimal toxicity and is effective in local tumor reduction, and it could further be considered for future treatment in small cutaneous metastases.

### 2.4. Electroporation and Immunotherapy

Immunotherapy is becoming an attractive cancer treatment also for HNSCC and recurrent and/or metastases H&N cancers [26]. Many approaches have been reported and evaluated; these include checkpoint inhibition, T cell transfer therapy, monoclonal antibodies, and cancer vaccination; however, high doses of immune therapeutic agents still caused some side effects [47]. Combining EP with immunotherapy is possible to achieve treatment dose reduction obtaining a synergic effect between EFP and therapy. The latter has, therefore, the ability to modulate the immune system to produce immune cytokines and agents in the patient’s body, increasing the cellular uptake of these immune agents via electroporation through cancer cell membranes.

The synergic effect of EP immune-activation and immunotherapy was tested. EP efficacy in reducing tumor size by initiating a host immune reaction against cancer cells was proved in immune-competent mice in contrast with immune-deficient mice, which did not give any improvement [123].

Nanosecond electrical field pulses (NsEFPs), a type of stimulation using electrical pulses which last only a few hundred nano seconds (30 kV/cm, 100 ns, 200 p), showed an inhibitory effect on proliferation of malignant melanoma promoting activation of immune cells (killer T cells) and increasing the release of anti-tumoral cytokines (TNF-α and IL-2). Further investigation could be performed by combining physical therapy with immunotherapy [124].

ECT was implemented with monoclonal antibody to enhance its antitumoral activity; preclinical evidence suggests that the association of ECT with immune-stimulating agents can be an efficient way to cure the targeted malignant tumors and any distant nontargeted tumor nodules, even if it is an undetectable metastasis [75,125].

ECT with ipilimumab was used to treat metastatic melanoma, and the results obtained showed a complete cutaneous, and visceral response of the 28 tumor nodules treated [126,127].

Ipilimumab (CTLA-4 inhibitor) and Nivolumab (PD-1 inhibitor) were tested in combination with ECT, and the results demonstrated that ECT + PD-1 inhibitors were more effective [128,129]. Pembrolizumab (PD-1 inhibitor) was used in combination with ECT in patients with unresectable melanoma with superficial and visceral metastases [130]. The multicenter study showed that the local objective response rate (ORR) was higher in the pembrolizumab-ECT group than in the pembrolizumab group (78% and 39%, *p* < 0.001). Moreover, 1-year follow-up showed that the one-year local progression-free survival (LPFS) rates were 86% and 51% (*p* < 0.001), respectively.

Table 3 reports the clinical trials that involved electroporation in the treatment of H&N cancers and correlated metastases.

### 2.5. Electroporation and Nanotechnology

Nanotechnology is an emerging field that involves the functionalization and engineering of nanosized materials. The National Nanotechnology Initiative (NNI, 2010) describes nanotechnology as “the understanding and control of matter at dimensions between approximately hundred nanometers, where unique phenomena enable novel applications” [131]. Thanks to their large surface area, nanomaterials (NMs) interaction with cells is maximized, increasing therapeutic effectiveness compared to traditional methods [132]. The result adds up to a decreased risk to the patient and an increased probability of survival [133]. NMs designed for cancer therapy are of various types, such as micelles, liposomes, dendrimers, inorganic nanoparticles (gold, silver, iron), carbon nanoparticles and nanotubes, nanodiamonds, nanoemulsions, viral nanocarriers, polymeric or peptide nanoparticles, and solid lipid nanoparticles; they can be used as stand-alone cancer therapies or can be used as adjuvants or as part of a combinatorial therapy [134]. Generically, these NMs can be called nanovectors (NVs), a type of targeted delivery vehicle (nanopharmaceutics and bioinspired nanoparticles) that transports nanoscale material [135].

Despite their small dimension, nanomaterials can easily reach tumor sites by exploiting the enhanced permeability and retention (EPR) effect (passive targeting) and protein coronas (PCs). EPR exploit characteristics of newly formed tumor vessels having poorly aligned and defective endothelial cells with wide fenestrations that allow passage of NVs and macromolecules [136].

PCs involve protein absorption onto particle surfaces, causing a different biological identity of NVs, which can cause two types of responses: immune blinding (reducing cancer uptake) or immune reactivity (excessive immune activity with increasing proinflammatory cytokines production). A Red blood cell (RBC) membrane coating, PEGylation, or the biocompatible materials used, such as zwitterionic polymers or hydrophilic nanoparticles, are strategies that can be used to decrease protein adsorption and thus avoid unwanted responses [137,138]. Furthermore, the advantages of the use of nanotechnology in cancer treatment are the ability to target chemotherapies directly and selectively (active targeting) to cancerous cells and neoplasms, guide cancer surgical resection, and enhance the therapeutic efficacy of radiation-based and other current treatment modalities. NMs can be functionalized on their surface with ligands (i.e., small molecules, peptides, fluorophores, antibodies) useful to (i) selectively direct NMs in vivo, (ii) exert therapeutic action, and/or (iii) act as imaging agents. These multiple combinations of actions, such as drug delivery for treatment and imaging detection for diagnosis, are known as “theranostic” actions [139]. Moreover, depending on NM physico-chemical properties is possible to modulate their activity (drug release, energy absorption, re-radiation, and localization) using external triggers such as electric and magnetic fields, hyperthermia, light, and ultrasound [140].

The Nanomedicine Strategic Research and Innovation Agenda (2016–2030) from the European Nanomedicine Community listed the most needed implementation for cancer treatment. These innovations concern the improvement of early detection diagnosis methods for tumors, circulating tumor cells, and metastases, maximizing the effectiveness of treatment for solid tumors and chemo-resistant tumors, making more selective and effective radiotherapy, immunotherapy, photodynamic, individualized, and hyperthermia therapies and avoid side effects applying more targeted chemotherapy [141].

NMs in H&N cancer treatment have the potential to emerge as alternatives to conventional treatments, as these systems can offer solutions (non-invasively, minimize non-specific delivery failures, reduce multidrug resistance) to the problems encountered in conventional treatments (chemotherapy or radiotherapy) [142,143,144]. The combination of EP with nanomedicines is starting to present itself as a valid adjuvant strategy for the treatment of some diseases, including liver, pancreatic, and bone tumors [145,146]. NVs combined with EP were tested in vitro for H&N cancer treatment. Gold nanoparticles (4.79 μg/ml of AuNPs) were used during electroporation (10 pulses at 200 V, equal time intervals of 4 sec) of Hep-2 laryngeal cancer cells inducing cell apoptosis, alterations of cell cycle profile, and morphological changes [147].

**Figure 5:**
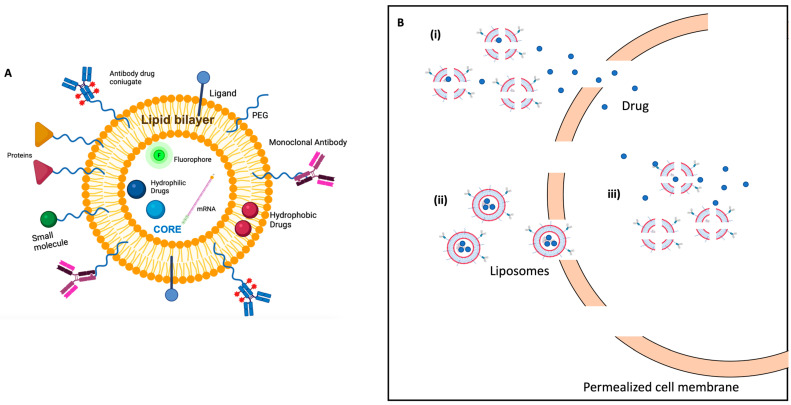
(**A**) graphic representation of lipid bilayer liposome carrying hydrophilic molecules in the core and hydrophobic molecules in the lipid portion with surface functionalization for selective, therapeutic, and/or diagnostic target action; (**B**) Electric field pulses effects on liposomes and cell membranes (i) liposomes membrane permealization and drug release nearby cell; (ii) liposome facilitated entry through permealized cell membrane; (iii) liposomes membrane permealization and intracellular drug release (created with BioRender—www.BioRender.com).

Nowadays, liposomes are the most successful drug delivery systems, with a dozen drug products available in clinics and FDA-approved cancer therapy (e.g., Doxil) [148,149]. Liposomes are self-assembled bilayers of lipid vesicles that possess an aqueous core and a hydrophobic membrane; this peculiar property allows them to encapsulate both hydrophilic and hydrophobic drugs. Depending on lipid bilayer structure, liposomes can be classified into small unilamellar vesicles (SUV), large unilamellar vesicles (LUV), and giant unilamellar vesicles (GUV) that have a single lipid bilayer, while multilamellar vesicles (MLV) are characterized by more than one lipid bilayer. Proper lipid selection is essential to modulate liposomes’ pharmacologic activity in such a way that. Zwitterionic, cationic, or anionic lipids and/or cholesterol have a different effect on liposome stability, pharmacokinetics, and delivery of the drug formulation [150]. Further functionalization of liposome surface, such as grafting poly-(ethylene glycol) (PEG), is useful to prevent protein binding and prolong liposomes blood circulation meanwhile avoiding uptake from the reticuloendothelial system (RES). Moreover, synthetic modification of the terminal PEG molecule with ligands (e.g., monoclonal antibodies and peptides) can be carried out to promote the selective and enhanced accumulation of liposomes in the tumor region with respect to healthy tissues [151,152]. Therefore, liposomes are very versatile drug delivery systems that can achieve passive or active drug targeting depending on their designed formulation (Figure 5A).

Liposomes are considered biocompatible due to their similarity with composition of biological membranes, low immunogenic, easily modifiable, reproducible, and scalable, and possess a good safety profile [153]. Clinical trials demonstrated that liposomal formulations are less toxic than drugs alone and have better pharmacological parameters [154]. They represent the first-choice drug delivery systems for various diseases. As a matter of fact, liposomes are under evaluation in clinical trials also for H&N cancer treatment (Table 4).

The first generation of traditional liposomes was commonly formulated using phospholipids and cholesterol. The most recent generation of lipid vesicles also includes the use of (i) surface functionalization to reach the specific cell or tissue (targeted therapy), (ii) adjustable and adaptive structure to be administered via transdermal and/or oral routes (transfersome) and, (iii) external stimuli sensitive liposomes (electric and/or magnetic field, ultrasound, UV/light) or internal stimuli sensitive (pH, temperature, redox potential, enzymes, electrolyte concentration) to achieve a spatiotemporal control of drug release (smart delivery system) [155,156,157].

Among smart delivery systems, liposomes and their response to electric pulse (electro-sensitive smart delivery systems) attract a lot of interest in combination with EP treatment to obtain a drug release on-site and on-demand [158,159]. Due to the similarity of liposomes and cell membrane phospholipidic bilayer, using EFPs is possible to induce both cells and liposomes membranes electroporation without triggering irreversible damage to cells. Therefore, when combining nanotechnology-based solutions, the EP goal is to design molecular carriers (e.g., liposomes) of nanoscale dimension (hundreds of nm) able to guarantee an intracellular or extracellular (close to the target cells) drug-controlled release by application of the external electric field. Liposome poration could permit a fast drug’s release in the intracellular medium and/or in the extracellular medium closer to the cells and an easy drug uptake by the electroporated cells (the concept is similar to ECT, with the benefit of increased drug concentration in the tumor due to its vectorization in liposomes) [160,161,162] (Figure 5B). This allows the development of a selective and targeted delivery system where the drug is activated only in the diseased site in the body (e.g., a cancerous tissue), avoiding any damage or toxicity to healthy cells and to the surrounding tissues.

The most important parameters that influence liposome behavior when subjected to an in vitro electric field are (i) cholesterol ratio, (ii) liposome surface charge, and (iii) liposome size.

Cholesterol is an important component for liposome formation and stability, and it was demonstrated that it has a concentration-dependent effect on lipid membrane organization. Raffy et al. evaluated cholesterol amount on phosphatidylcholine bilayer stability under imposed electric field [163]. The study was performed on lipids in gel (1,2-dipalmitoyl-sn-3- phosphatidylcholine-DPPC) and in fluid states (egg 3-phosphatidylcholine-PC). For lipids in the gel state, cholesterol in a concentration equal to 6% (mol/mol) prevents electropermeabilization, while for concentrations higher than 12% (mol/mol), electropermeabilization and electroinsertion are obtained under milder field conditions (0.3 kV/cm). Instead, cholesterol does not affect electro-permeabilization and electro-insertion in lipids in the fluid state.

It is well known that liposomes’ surface charge (positive, neutral, or negative) influences their permeation. For example, Ogiso et al. demonstrated higher skin penetration of negatively charged liposomes [164]. Conversely, liposomes having positive charge are readily taken up by the cells due to electrostatic attraction between liposomes and negatively charged cell membrane [165]. In terms of stability and pores formation under electric field stimulation, negatively charged liposomes (i.e., POPS-1-palmitoyl-2-oleoyl-sn-glycerol-3-phospho- L-serine) allow greater ion transport by forming large hydrophobic pores compare to positively charged (i.e., POPC-1-palmitoyl-2-oleoyl-sn-glycerol-3-phosphatidylcholine) [166,167].

Concerning their size, liposomes >500 nm can cause an immune system reaction in the body but have dimensions more similar to cells; they require the same electric field amplitude for electroporation. Instead, liposomes <500 nm show higher cell internalization (due to the EPR effect) and low immunogenicity, but they require higher field values to be permeabilized, which could cause irreversible electroporation and cell death. Schwan’s equation at the steady-state explains this direct proportionality between transmembrane voltage (Vm) and particle radius (Equation (1)).

The strategy to overcome transmembrane dependence on the radius of microscopic structures for pulses with a lower frequency spectral content was the application of a second-order model of induced transmembrane potential (TMP) exploiting nanosecond pulses at higher intensity MV/m (NsEFPs) [162,168].

Denzi et al. explored NsEFPs applicability for remote control of electro-sensitive smart delivery systems in order to achieve electro-permeabilization of liposome membrane (100, 200, and 400 nm and membrane thickness 5 nm) with electric field amplitudes similar to the ones needed to simultaneously permeabilize a biological cell membrane [161]. Using unilamellar liposomes (200 nm) and 12 NsEFPs, they demonstrated that it is possible to permeabilize both liposomes and cells with comparable electric field intensity without damaging cells (10% of cell membrane area was porated). The results obtained confirmed that the difference in cells and liposome dimensions is not so crucial when using NsEFPs [161,162].

Caramazza et al. studied NsEFPs liposome activation in dry and wet experiments. A fluorescent dye was used as a drug model to visualize its release after electric field stimulation (10 ns, 14 MV/m, 2 and 4 Hz). In total, 15–20% drug release was achieved after a single treatment, so a multi-dose NsEFPs can be potentially used in the future to improve the amount of drug released. Moreover, in their work, the authors considered the effect of NsEFPs’ interaction with liposomes in terms of electromagnetic energy absorption, temperature distribution, and pore density formation [159].

Retelj et al. evaluated NsEFPs’ ability to control intracellular drug release from liposomes (50–500 nm). The aim was to obtain an efficient electro-sensitive drug release keeping the plasma and nuclear membranes of cells intact. The results achieved showed that using shorter pulses (10 ns) and larger liposomes, the possibility of selective electroporation is higher, with smaller risks for cell viability. Liposomes with 500 nm could be electroporated using ≈20 KV/cm, while 50 nm liposomes required higher amplitude >150 kV/cm. Moreover, liposomes with higher internal conductivity and lower membrane permittivity are favorably electroporated using NsEFPs. Instead, liposomes’ location inside the cell did not influence liposomes electroporation, so liposomes having similar sizes are electroporated simultaneously [160].

Tian et al. demonstrated in vitro antitumor activity of liposomes loaded with a dual PI3 K/mTOR inhibitor (NVP-BEZ235) in combination with irreversible electroporation (IRE) for H&N cancer treatment. After 1 month, in the nude mice model, only a combination with irreversible EP and loaded liposome was able to eradicate tumor masses, demonstrating that this combination of treatments could be useful to eradicate cancer and prevent recurrence [169].

These demonstrations would open the way to a feasible use of nano-pulses for electro-sensitive smart drug delivery applications where the electric pulse acts as a drug release remote controller by the carrier (i.e., liposomes) and as a facilitator of drug internalization in the cell.

## 3. Conclusions and Future Prospective

The incidence rate of H&N cancers is likely to increase, and especially recurrent and metastatic forms are the most frequent and difficult to treat. Improved antitumoral treatments are an important tool in order to counteract this increase in aggressive forms and improve the patient’s quality of life. In this regard, the use of nanomedicine and medical technologies has greatly shown their clinical potential in cancer treatment. Interestingly, the use of nanovectors (nanopharmaceutics and bioinspired nanoparticles) demonstrated higher therapeutic potential in the treatment of different types of cancers, including H&N, as they increase target selectivity, attenuate drug toxicity, and protect drugs from rapid clearance [51,135,170].

In this review, the latest innovations in the treatment of H&N cancer exploiting EP were summarized and discussed. Reversible EP can be considered a safe and effective technique able to act synergically, permeabilizing cancer cell membranes and, at the same time, inducing functional vasoconstriction and host immune system activation. Currently, ECT with Bleomycin is an emerging treatment modality for the treatment of superficial and cutaneous metastatic cancer, and its improvement using immunotherapy and/or nanotechnology approaches is to be considered and evaluated. The potentiality of EP and ECT as advanced treatments was preliminarily investigated for those exploiting nanotechnology-based or immunotherapy solutions.

Many clinical trials have already started to test the synergistic activity of electrochemotherapy with monoclonal antibodies and the action of liposomal nanocarriers against H&N cancers. Moreover, the effect of the electric field applied to lipid nanosystems for the on-site and on-demand control of drugs released in vitro is well described in the literature and awaits practical findings in the clinic.

## Figures and Tables

**Table 1:** Molecular biomarkers for HNSCC and CSCs.

Biomarker		Activity	Ref.
EGFR	Epidermal growth factor receptor	Controlling gene expression, proliferation, angiogenesis, apoptosis inhibition, cell motility, metastasis, adhesion, and angiogenesis	[26]
CD44	Surface receptor for hyaluronic acid and matrix metalloproteinases (MMPs)	Intercellular interactions and cell migration	[27]
CD133	Transmembrane glycoprotein	Invasiveness and metastasis	[28,29]
ALDH1	Intracellular enzyme able to convert retinol into retinoic acid Cellular detoxification	Marker for both normal stem cells and CSCs	[25]
STAT3	Protein transcription factor	It drives expression of genes promoting cellular proliferation and survival and genes encoding growth factors and cytokines promoting immunosuppression (IL-6, IL-10, and TGF-beta)	[30]
PTPRs	Protein tyrosine phosphatase receptors	It causes STAT3 hyperactivation in H&N	[31]
PD-L1	Programmed death-ligand transmembrane protein	Biding receptor PD-1 suppresses the adaptive immune system	[32]

**Table 2:** Monoclonal antibodies currently used in HNSCCs treatment.

Monoclonal Antibody	Commercial Name	Mechanism of Action	Clinical Indication	Note
Cetuximab	Erbitux^®^; Merck	Binds with high affinity to the extracellular domain of human EGFR inhibiting receptor activity targets cytotoxic immune effector cells towards EGFR-expressing tumor cells (antibody-dependent cell-mediated cytotoxicity)	Patients with recurrent or metastatic disease Cisplatin-ineligible patients	FDA and EMA approved for HNSCC [36]
Pembrolizumab	KEYTRUDA^®^, Merck	Targeted programmed cell death protein PD-L1 (immune checkpoint inhibitors)	Cisplatin-sensitive HNSCC Patients with metastatic or unresectable recurrent HNSCC	FDA; EMA approved for HNSCC [37]
Nivolumab	Opdivo ^®^ Bristol-Myers Squibb	Binds to the PD-1 receptor and blocks its interaction with PD-L1 and PD-L2 (immune checkpoint inhibitors)	Cisplatin-refractory recurrent or metastatic HNSCC	FDA and EMA approved for cancer treatments and in combined therapy for HNSCC [38]
Ipilimumab	Yervoy ^®^ Bristol-Myers Squibb	Binds CTLA-4 inhibitory signal, activating immune system	Loco-regionally advanced HNSCC	Evaluated in combination with HNSCC treatment [38]. Not yet approved by EMA

**Table 3:** Clinical trials (www.clinicaltrials.gov) that involved electroporation in the H&N cancer treatment.

NCT Number	Title	Interventions	Phase	Last Update	Location
NCT03051269	Calcium electroporation for head-and-neck cancer	Drug: calcium chloride device: electroporation	1	2017	Department of Otorhinolaryngology, Rigshospitalet, Copenhagen University Hospital, Copenhagen, Denmark
NCT02549742	Electrochemotherapy on head-and-neck cancer	Procedure: Electrochemotherapy Device: Cliniporator •Drug: bleomycin	2	2017	Department of Otorhinolaryngology, Rigshospitalet, Copenhagen University Hospital, Copenhagen, Denmark
NCT00198315	Medpulser Electroporation with Bleomycin Study to treat anterior head-and-neck squamous cell carcinoma	Combination product: Medpulser electroporation with bleomycin Procedure: tumor surgical excision	3	2017	Inovio Biomedical Corporation, San Diego, California, United States
NCT00198263	Study using the Medpulser electroporation system with Bleomycin to treat head-and-neck cancer	Combination product: Medpulser electroporation with bleomycin	4	2017	Inovio Biomedical Corporation, San Diego, California, United States
NCT01493154	Safety study of HPV DNA vaccine to treat head-and-neck cancer patients	Biological: DNA vaccine drug: cyclophosphamide	1	2018	Sidney Kimmel Comprehensive Cancer Center, Johns Hopkins Hospital, Baltimore, Maryland, United States
NCT02960594	hTERT immunotherapy alone or in combination with IL-12 DNA followed by electroporation in adults with solid tumors (H&N cancer and esophageal cancer) at high risk of relapse (TRT-001)	Biological: INO-1400 Biological: INO-9012 Biological: INO-1401	1	2018	Barbara Ann Karmanos Cancer Institute Detroit, Michigan, United States Mayo Clinic Rochester, Minnesota, United States And more.
NCT02345330	Trial of pIL-12 electroporation in squamous cll carcinoma of the head and neck (IL12HNSCC)	Biological: Tavokinogene Telseplasmid (tavo) Device: OncoSec Medical System (OMS)	2	2018	UCSF Helen Diller Family Comprehensive Cancer Center, San Francisco, California, United States University of Chicago Medical Center, Chicago, Illinois, United States
NCT01941901	Calcium electroporation for treatment of cutaneous metastases	Drug: calcium electroporation Drug: electrochemotherapy with bleomycin	2	2019	Department of Oncology, Copenhagen University Hospital, Herlev, Herlev, Denmark
NCT03823131	Optimizing antitumor immunity using plasmid electroporation, Pembrolizumab, and Epacadostat	Device: ImmunoPulse Drug: Epacadostat Drug: Pembrolizumab Biological: CORVax Drug: Tavokinogene telseplasmid	2	2021	University of California, San Francisco, San Francisco, California, United States
NCT02163057	Study of HPV-specific immunotherapy in participants with HPV-associated head-and-neck squamous cell carcinoma	Biological: INO-3112 •Device: CELLECTRATM-5P	2	2021	University of Pennsylvania, Philadelphia, Pennsylvania, United States
NCT03448666	ECT-Pembrolizumab in patients with unresectable melanoma with superficial or superficial and visceral metastases	Combination product: Pembrolizumab	2	2021	IEO Istituto Europeo di Oncologia, Milan, Italy
NCT03162224	Safety and efficacy of MEDI0457 and Durvalumab in patients with HPV associated recurrent/metastatic head-and-neck cancer	Drug: MEDI0457 Device: CELLECTRA^®^5P device (CELLECTRA 2000) Drug: Durvalumab	2	2021	San Francisco, California, United States Orlando, Florida, United States Atlanta, Georgia, United States Indianapolis, Indiana, United States Baltimore, Maryland, United States Baltimore, Maryland, United States And more.

**Table 4:** Liposomes under evaluation in clinical trials (www.clinicaltrials.gov) for the treatment of H&N cancers (Keywords used for the search as head-and-neck cancer and liposomes).

NCT Number	Title	Interventions	Phase	Last Update	Location
NCT00252889	Doxil Topotecan doublet cancer Study (H&N cancer, Esophageal cancer)	Drug: Topotecan and pegylated doxorubicin	1	2009	Christiana Care Health Services, Newark, Delaware, United States
NCT00022594	Liposomal Lurtotecan in treating patients with metastatic or locally recurrent head-and-neck cancer	Drug: liposomal lurtotecan	2	2012	Kaiser Franz Josef Hospital, Vienna (Wien), Austria Universitair Ziekenhuis Antwerpen, Edegem, Belgium Centre Jean Perrin, Clermont-Ferrand, France And more
NCT00009841	Gene therapy in treating patients with advanced head-and-neck cancer	Biological: EGFR antisense DNA Biological: growth factor antagonist therapy Drug: DC-cholesterol liposome	1	2016	University of Pittsburgh Cancer Institute, Pittsburgh, Pennsylvania, United States
NCT02262455	Effects of STM 434 alone or in combination with liposomal doxorubicin in patients with ovarian cancer or other advanced solid tumors (H&N cancer)	Drug: STM 434 (inhibitor of activin A) Drug: liposomal doxorubicin	1	2017	Dana Farber Cancer Institute Boston, Massachusetts, United States Memorial Sloan Kettering Cancer Center New York, New York, United States And more
NCT03076372	A study evaluating MM-310 in patients with solid tumors (HNSCC)	MM-310 is a liposomal formulation of a docetaxel	1	2018	Honor Health, Scottsdale, Arizona, United States University California San Francisco, San Francisco, California, United States Mayo Clinic, Rochester, Minnesota, United States
NCT00006036	Liposomal Lurtotecan plus Cisplatin in treating patients with advanced or metastatic solid tumors	Drug: cisplatin Drug: lurtotecan liposome	1	2020	British Columbia Cancer Agency, Vancouver, British Columbia, Canada Cancer Care Ontario-Hamilton Regional Cancer Centre, Hamilton, Ontario, Canada Toronto General Hospital, Toronto, Ontario, Canada
NCT04902027	A study of Mitoxantrone Hydrochloride liposome injection in the treatment of recurrent/metastatic head-and-neck cancers	Drug: mitoxantrone hydrochloride liposome, intravenous injection (IV)	1	2021	-
NCT04244552	A Phase 1b trial of ATRC-101 in adults with advanced solid malignancies (HNSCC)	Biological: ATRC-101(engineered fully human immunoglobulin G, subclass 1 (IgG1) antibody) Biological: Pembrolizumab Drug: pegylated liposomal doxorubicin (PLD)	1	2021	Mayo Clinic Phoenix, Arizona, United States The University of Arizona Cancer Center Tucson, Arizona, United States And more

## Acronyms

ADCC	Antibody-dependent cell cytotoxicity
APCs	Antigen-presenting cells
BLM	Bleomycin
Ca^2+^	Calcium
CisPt	Cisplatin
CRT	Chemoradiotherapy
CSCs	Cancer stem cells
DAMPs	Damage-associated molecular patterns
ECT	Electrochemotherapy
EFPs	Electrical field pulses
EMA	European Medicines Agency
EP	Electroporation
EPR	Enhanced permeability and retention effect
ER	Endoplasmic reticulum
FDA	U.S. Food and Drug Administration
FU	5- fluorouracil
GA	General anesthesia
GE	Gene electroporation
Gy	Gray
H&N	Head and neck
HNSCC	Head-and-neck squamous cell carcinoma
HPV	Human papillomavirus
K	Dielectric constant
IL	Interleukin
I.T.	Intra-tumor
I.V.	Intra-venous
LA	Local anesthesia
MCAM	Melanoma cell adhesion molecule
NMs	Nanomaterial
NV	Nanovector
NsEFPs	Nanosecond electrical field pulses
OS	Overall survival
PCs	Protein coronas
PD-L1	Programmed death ligand 1
PEG	poly-(ethylene glycol)
QoL	Quality of life
RB1	Retinoblastoma-associated protein
RT	Radiotherapy
SOP	Standard operating procedure
SR	Sarcoplasmic reticulum
TMP	Transmembrane potential
Vm	Transmembrane voltage
